# Nutritional status in HIV-infected patients using Changi's mini nutritional assessment

**DOI:** 10.1186/1758-2652-13-S4-P76

**Published:** 2010-11-08

**Authors:** M Cervero, V Alcázar, R Sanz, C García-La Calle, JL Agud

**Affiliations:** 1 H. Severo Ochoa, Internal Medicine, Leganés, Spain; 2 H. Severo Ochoa, Endocrinology Service, Leganés, Spain; 3 H. Severo Ochoa, Laboratory Service, Leganés, Spain

## Objective

The achievement of an adequate nutritional status is an important goal in the treatment of HIV infection and requires a proper assessment. We studied the nutritional status of a group of HIV-infected patients using biochemical and anthropometric measures.

## Materials and methods

We assessed 91 outpatients followed in an urban infectious disease clinic in south Madrid (Spain). In each case age, gender, race, HIV risk group, hepatitis C virus (HCV) infection status, smoking status, degree of immune depression, viral load, presence or absence of an AIDS diagnosis, time since diagnosis of HIV infection, antiretroviral therapy (ART), type of ART, anthropometric measures (weight, waist circumference, BMI, tricipital fold and arm circumference), and blood and biochemical measures (lymphocyte count, CD4+ cell count, albumin, triglycerides, cholesterol).

## Results

Median age was 46 years; 69.2% were males, 81.3% Caucasian. Viral load was below 20 copies/ml and mean CD4 count 468 cells/µL Using Changi's mini nutritional assessment, which involves immunological, biochemical and anthropometric parameters, we found malnutrition in 81.1%; it was severe degree in 16.7%. Most patients were classified as caloric malnutrition (67.8%). Serum cholesterol was increased in 24.7% and triglyceride in 33.3%. Serum albumin was normal in 96.6%.

Anthropometric measurements showed diminished fat compartment (decreased tricipital fold in 66.7% of males and 51.9% of females) whereas muscular compartment was preserved (muscular arm circumference was decreased in 34.9% of males and 25.9% of females). Tricipital fold 50th percentile was 8 mm in males and 19.9 mm in women; corresponding values for arm circumference were 26 cm and 22 cm, respectively. Of males, 6.6% were underweight, as were 23.1% of women. Malnutrition was associated with BMI (p=.0001), waist circumference (p=.029) and with HCV infection (p=.037). Caloric malnutrition was associated with BMI (p=.0001), waist circumference (p=.046) and chronic HCV infection (p=.049). It was also related with the degree of immune suppression (<200, 200-500 and >500 CD4+ cells/µL; p=.019).

## Conclusions

Despite HAART we found malnutrition, at least of small degree, in more than 80% of patients, mainly caloric malnutrition. Arm fat compartment was most frequently involved. Chronic HCV infection, degree of immune suppression, low BMI and waist circumference are related with the presence of malnutrition.

